# Metagenomic mining for thermostable esterolytic enzymes uncovers a new family of bacterial esterases

**DOI:** 10.1038/srep38886

**Published:** 2016-12-19

**Authors:** Dimitra Zarafeta, Danai Moschidi, Efthymios Ladoukakis, Sergey Gavrilov, Evangelia D. Chrysina, Aristotelis Chatziioannou, Ilya Kublanov, Georgios Skretas, Fragiskos N. Kolisis

**Affiliations:** 1 Institute of Biology, Medicinal Chemistry & Biotechnology, National Hellenic Research Foundation, Athens, Greece; 2 Laboratory of Biotechnology, School of Chemical Engineering, National Technical University of Athens, Athens, Greece; 3 Winogradsky Institute of Microbiology, Research Center for Biotechnology Russian Academy of Sciences, Moscow, Russian Federation

## Abstract

Biocatalysts exerting activity against ester bonds have a broad range of applications in modern biotechnology. Here, we have identified a new esterolytic enzyme by screening a metagenomic sample collected from a hot spring in Kamchatka, Russia. Biochemical characterization of the new esterase, termed EstDZ2, revealed that it is highly active against medium chain fatty acid esters at temperatures between 25 and 60 °C and at pH values 7–8. The new enzyme is moderately thermostable with a half-life of more than six hours at 60 °C, but exhibits exquisite stability against high concentrations of organic solvents. Phylogenetic analysis indicated that EstDZ2 is likely an *Acetothermia* enzyme that belongs to a new family of bacterial esterases, for which we propose the index XV. One distinctive feature of this new family, is the presence of a conserved GHSAG catalytic motif. Multiple sequence alignment, coupled with computational modelling of the three-dimensional structure of EstDZ2, revealed that the enzyme lacks the largest part of the “cap” domain, whose extended structure is characteristic for the closely related Family IV esterases. Thus, EstDZ2 appears to be distinct from known related esterolytic enzymes, both in terms of sequence characteristics, as well as in terms of three-dimensional structure.

Lipolytic enzymes (EC 3.1.1.x) hydrolyze the ester bonds in lipids and have received great attention due to their potential use in a broad range of biotechnological applications, such as the production of biofuels and fine chemicals^1^,^2^. Depending on whether they hydrolyze small water-soluble esters or ester bonds of long-chain acylglycerols, they are classified as carboxylesterases (EC 3.1.1.1) or lipases (EC 3.1.1.3)^3^. Both types of enzymes are serine hydrolases that contain a functional serine at their active site, which is usually located within the conserved sequence motif G-X-S-X-G^4^. This serine, along with an acidic residue -aspartic acid or glutamic acid- and a histidine, to which the acidic residue is hydrogen-bonded, constitute a highly conserved catalytic triad^5^. In addition, ester hydrolases share common structural features, such as a characteristic α/β hydrolase fold, as well as biochemical characteristics, like co-factor independence^3^,^6^.

Based on amino acid sequence homology and fundamental biochemical properties, bacterial lipolytic enzymes were initially classified into eight distinct families in the landmark paper by Arpingy and Jaeger^5^. Family I consists of lipases and includes six subfamilies^5^. The remaining seven families were denoted as Families II-VIII and include carboxyl esterases or “true esterases”. Despite the fact that this classification was proposed for bacterial esterases, it should be noted that many families in the original analysis included not only bacterial sequences, but also archaeal and eukaryotic representatives^5^. The more recent discovery of the lipase-like poly[(R)-3-hydroxybutyrate] depolymerase PhaZ7^7^, the hyperthermophilic esterase EstD^8^, and a large number of additional metagenome-derived esterolytic hydrolases^9^,^10^,^11^,^12^, expanded this classification to include six new families, denoted as Families IX-XIV. More recently conducted metagenomic studies have revealed a plethora of new bacterial lipolytic enzymes that seem to define additional new families, which do not follow the ordered numerical indicies of the original Arpingy and Jaeger classification. Examples of such recently discovered families of bacterial esterolytic enzymes are the ones defined originally by the esterases Est10^13^, EstWSD^14^, EstD2^15^, and EstLiu^16^, among others.

Carboxyl esterases and lipases are valuable industrial enzymes. Their native hydrolytic nature is complemented by a broad range of substrate specificities, as well as high stereo- and regio-selectivity^17^. Besides their physiological role, lipolytic enzymes possess the ability to perform synthesis of esteric bonds or transesterification reactions in non-aqueous media, following the thermodynamic reversion of the hydrolysis reaction route^18^. These features render them protagonist biocatalysts in the flavor and aroma, food, biodiesel, fine chemical, cosmetics, detergent, and pharmaceutical industries^19^,^20^,^21^. Their biotechnological significance is reflected by the fact that several esterases and lipases have been commercialized by leading biotech companies such as Fluka, Novozymes, Amano, Diversa, Roche Diagnostics, Thermogen and others^4^.

Nowadays, an important bottleneck to the wide industrial use of lipolytic enzymes is that they are often required to perform under harsh conditions. These include temperatures above 40 °C, significant concentrations of organic solvents, metal ions, surfactants, and other agents known to promote enzyme denaturation and inactivation^22^,^23^,^24^. As a result, stability against elevated temperatures and overall tolerance to protein-destabilizing conditions is a crucial prerequisite before the broad industrial use of this type of enzymes is realized.

Lipolytic enzymes are ubiquitous in nature and they can be found in all three domains of life (bacteria, archaea, eukarya) residing in all types of environmental niches, including high-temperature ecosystems. Their abundance has led to the discovery and characterization of a number of thermostable esterases and lipases, the majority of which originate from cultivable thermophilic microorganisms^6^. While the need for novel enzymes is ever-growing, the biocatalytic capabilities of the vast majority of microbial organisms remain unexplored due to the fact that more than 99% of them cannot be cultured using standard laboratory procedures^25^,^26^. Metagenomic approaches aim to address this issue and bypass the limitations imposed by current culturing techniques. This is achieved by providing direct access to the genomic material of microorganisms residing in extreme environments, where biocatalysts resistant to harsh “industrial conditions” are likely to be encountered. Furthermore, besides a discovery tool for biotechnology-relevant enzymes, metagenomic analysis enhances our understanding of the physiology of microbial communities residing in different types of environments, their complex relationships, and the related evolutionary paths^27^.

In this study, we aimed to identify new lipolytic enzymes with novel sequence and functional characteristics, and with properties suitable for industrial applications. To achieve this, we investigated the putative lipolytic enzymes encoded in the genomes of microorganisms residing in a hot spring located in Kamchatka, Russia. This site possesses characteristics, which are expected to favor the presence of organisms with various hydrolytic activities, including lipolytic ones. Environmental sampling from this location, followed by high-throughput sequencing of the isolated metagenomic DNA and bioinformatics screening for the identification of putative esterolytic sequences, revealed an open reading frame, which presented a low – but notable – similarity to previously characterized esterases. The identified sequence was amplified, cloned, expressed in *Escherichia coli*, and purified in soluble and functional form. Detailed biochemical characterization of this recombinant protein demonstrated that it constitutes a new carboxyl esterase with preference towards esters of fatty acids with medium chain length. The new enzyme, termed EstDZ2, was found to exhibit moderate thermostability, as well as significant tolerance against a variety of organic solvents, metal ions and other denaturing agents. Notably, phylogenetic analysis revealed that the sequence of EstDZ2 does not cluster with that of any other characterized bacterial esterase, but instead exhibits characteristics that deviate from those encountered in related families of bacterial esterolytic enzymes. In accordance with this, protein structural modelling suggested that some of the structural features that characterize Family IV esterases, the most closely related family of EstDZ2 and its homologs, appear to be absent from the structure of EstDZ2. EstDZ2, thus, constitutes a new candidate enzyme for industrial biotransformations, which appears to belong to a new family of esterolytic enzymes. For this new family of bacterial esterolytic enzymes, we propose the index XV.

## Results

### Environmental sampling

An environmental sample was collected from the Solnechny (Sun) hot spring located in Uzon Caldera, Kronotsky reserve, Kamchatka, Russia (coordinates: 54° 29’ 56” N, 159°59’ 31” E) in September 2011. At the moment when the sample was taken, the temperature of the site was 61–64 °C and the pH 5.8–6.0. Detailed description of the site is given by Menzel *et al*.^28^. The hot spring is located in a geothermal swamp and constitutes a small pool that emanates CO_2_. Its bottom is covered by a thick layer of plant residues buried in sludge and iron oxide depositions, and it is surrounded by stromatolites. It is, thus, characterized by a high content of organic carbon and active processes of organic matter degradation, conditions that should favor the presence of organisms with various hydrolytic activities, including lipolytic ones. A recent metagenomic study of the microbial diversity of the Solnechny hot spring revealed the prevalence of bacterial species, 40% of which could not be assigned to any phylum^28^. These characteristics make the Solnechny hot spring a highly promising source for identifying new enzymes with a broad range of activities and high levels of stability against denaturing conditions. Metagenomic analysis in this work was carried out using a sample collected from the mud, as this was found to be the richest part of the hot spring in terms of microbial diversity^28^.

### Discovery of EstDZ2

Metagenomic DNA was isolated from the aforementioned environmental sample and sequenced using a Roche/454 Titanium FLX high-throughput sequencing platform. Reads were assembled into contigs, and the predicted open reading frames (ORFs) were examined against NCBI’s conserved domain database^29^ for the presence of domains related to esterolytic activities, as described in the Methods section. From this analysis, we selected for further investigation a putative protein sequence, termed EstDZ2α, which was assigned to the esterase/lipase superfamily by NCBI’s conserved domain database and contained multiple esterolytic-like domains. The EstDZ2α sequence comprises 272 amino acids and has a predicted molecular mass of 29.4 kDa. Two sequence motifs, which are highly conserved among different families of esterolytic enzymes were identified: (i) the GXSXG motif, which is expected to be part of the active site, with the catalytic serine at the center, and (ii) the tetrapeptide sequence HGGG, which is known to contribute to the formation of the oxyanion hole required for catalysis in this class of enzymes^30^. Analysis using Delta-BlastP revealed 23% amino acid sequence identity (90% coverage) to a previously characterized isoprenylcysteine methylesterase from *Arabidopsis thaliana*^31^. Analysis of the EstDZ2α sequence using the SignalP 4.1^32^ and Signal-Blast^33^ servers, predicted that the first 21 amino acids of the protein serve as a signal peptide, thus suggesting that EstDZ2α is naturally a secreted enzyme.

**Figure 1:**
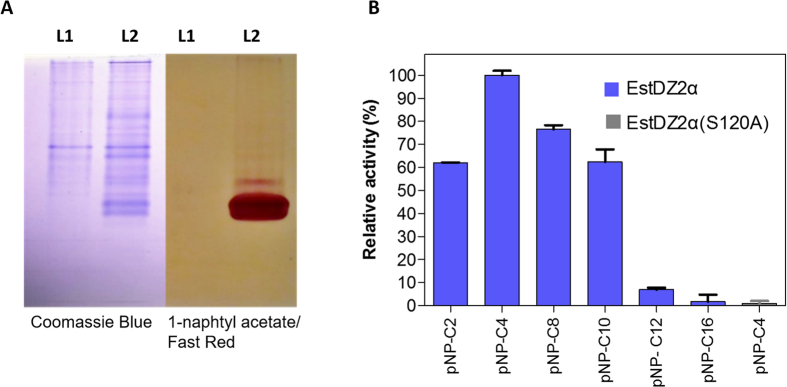
Discovery of the new esterolytic enzyme EstDZ2 and initial substrate specificity analysis. **(A)** Detection of EstDZ2α esterolytic activity via native PAGE analysis and Fast Red staining using 1-naphthyl acetate as a substrate. L1: Lysate of cells carrying an empty vector, L2: Lysate of cells overexpressing *estDZ2α*. **(B)** Initial substrate profiling of the esterolytic activity of EstDZ2α and the variant EstDZ2α(S120A). The relative enzymic activity was measured as released pNP at 410 nm (pH 7, 40 °C). The reported values correspond to the mean value of three independent experiments performed in triplicate and the error bars to one standard deviation from the mean value.

The *estDZ2a* gene was amplified by PCR from the metagenomic DNA sample and was cloned into the plasmid pET-28a(+) to form the vector pET-EstDZ2α. *E. coli* BL21(DE3) cells were transformed with pET-EstDZ2α, grown in 5 ml of Luria-Bertani (LB) medium at 37 °C with shaking until the culture reached an optical density at 600 nm (OD600) of 0.5, at which point 0.2 mM isopropyl thio-β-D-galactoside (IPTG) were added to induce *estDZ2a* overexpression. Τhese cells were collected, lysed by brief sonication and their lysates were assayed for their ability to hydrolyze *p*-nitrophenyl butyrate colorimetrically. We observed that the characteristic yellow color of *p*-nitrophenol (pNP), which is indicative of ester bond cleavage, appeared only when *estDZ2α* was expressed (see below), thus indicating that EstDZ2α possesses esterolytic activity against pNP-butyrate. Furthermore, when the proteins contained in the same cell lysates were separated by native polyacrylamide gel electrophoresis (PAGE), the gel was exposed to 1-naphtyl acetate as a potential substrate for hydrolysis, and stained with Fast Red, a band of red-brown color appeared rapidly after the addition of the substrate/indicator mixture only for the EstDZ2α-producing sample (Fig. 1A). Taken together, these results demonstrate that EstDZ2α possesses esterolytic activity.

An initial substrate preference test, using soluble lysates from EstDZ2α-producing cells and pNP-esters derived from fatty acids with a range of carbon chain lengths, demonstrated that this enzyme exhibits esterolytic activity against medium chain lengths (C2-C10), while its activity is barely detectable for C12 or larger substrates (Fig. 1B). To investigate the esterolytic activity of EstDZ2α against natural substrates, a variety of naturally occurring esters, such as ethyl-acetate, butyl-acetate, cinnamyl-acetate, tributyrin and triolein, as well as natural oils, such olive oil, palm oil, and sunflower oil, were tested. Esterolytic activity against these substrates was assayed using the pH indicator bromothymol blue, which detects the drop in the pH of the reaction that occurs due to the release of H^+^ ions upon ester bond hydrolysis^34^. Clarified lysates of *estDZ2α*-overexpressing cells caused a rapid color change in this assay only when tributyrin was added as a substrate (Supplementary Fig. S1). After long incubation times, a slight color change was also detected in the presence of cinnamyl-acetate, thus indicating that EstDZ2α might have very low activity against this substrate (data not shown). No activity against triolein and the tested natural oils could be detected even at long incubation times (Supplementary Fig. S1 and data not shown). These results are in agreement with the initial substrate specificity test using pNP-esters, where long-chained pNP esters could be not hydrolyzed by EstDZ2α and where the enzyme exhibited the highest levels of esterolytic activity against pNP-butyrate. Collectively, these results demonstrate that EstDZ2α is a new esterolytic enzyme that acts as an esterase rather than a lipase.

### Biochemical characterization

In order to study the biochemical properties of the newly identified enzyme, we produced it recombinantly in *E. coli* and purified it in soluble form. For this purpose, we cloned a truncated version of the *estDZ2a* gene, in which the predicted N-terminal signaling sequence of EstDZ2α (amino acids 2–21) was replaced with a hexa-histidine tag, and the resulting gene, referred to as *estDZ2*, was inserted again into pET-28a(+) to form plasmid pET-EstDZ2. Overexpression of *estDZ2* in *E. coli* BL21(DE3) resulted in the accumulation of primarily soluble enzyme with an apparent molecular mass of about 29 kDa under denaturing conditions, and the recombinant enzyme was purified via immobilized metal affinity chromatography to near homogeneity (Supplementary Fig. S2). Native PAGE, size exclusion chromatography and dynamic light scattering (DLS) analyses of EstDZ2 revealed that it functions as a monomer (Supplementary Fig. S3–S5).

**Figure 2:**
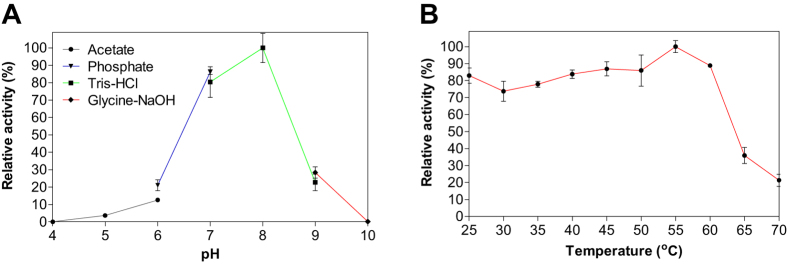
Effect of pH and temperature on EstDZ2 activity. **(A)** Effect of pH on EstDZ2 activity. Enzymic activity was measured in the standard assay reaction at 40 °C for 5 min, at pH values ranging from 4 to 10, using the indicated buffers. **(B)** Effect of temperature on EstDZ2 activity. Enzymic activity was measured at temperatures ranging from 25 to 70 °C and pH 8. The reported values correspond to the mean value from three independent experiments performed in triplicate and the error bars to one standard deviation from the mean value.

Detailed biochemical characterization of EstDZ2 was carried out as described in the Materials and Methods section, using pNP-butyrate as a substrate. First, we determined the optimal pH for EstDZ2 esterolytic activity, which was assayed within the pH range of 4–10 at 40 °C. Optimal activity for EstDZ2 was recorded at pH 7–8 (Fig. 2A). Below pH 7 and above pH 8, the esterolytic activity of EstDZ2 was rapidly diminished (Fig. 2A). Measurements of its relative catalytic activity at different temperatures, on the other hand, revealed that EstDZ2 has a broad temperature range of action, as its levels of esterolytic activity were practically unaltered at temperatures between 25 and 60 °C (Fig. 2B).

In order to investigate the esterolytic activity of EstDZ2 in more detail, we determined the catalytic parameters of EstDZ2-mediated hydrolysis for a range of pNP-esters derived from fatty acids with variable carbon chain lengths. Michaelis-Menten kinetics were observed for the hydrolysis of all substrates, which could be rapidly hydrolyzed by EstDZ2. In accordance with the initial substrate profiling study described above, EstDZ2 exhibited high catalytic rates (*k*_cat_) against middle- to long-chain fatty acid esters (C4-C10; Table 1). For C12 or larger substrates, pNP-ester hydrolysis was barely detectable. Catalytic efficiency was optimal for pNP-butyrate (C4) with a *k*_cat_/*K*_m_ value of 14,375 s^−1^mM^−1^ and decreased with increasing chain length for C8 and C10 (Table 1).

### Thermostability and effect of metals, detergents and organic solvents on the activity of EstDZ2

**Figure 3:**
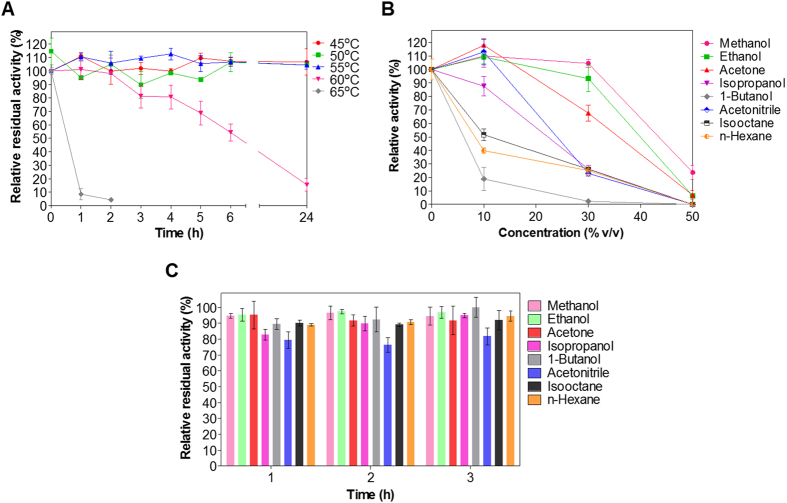
EstDZ2 thermostability and organic solvent tolerance and stability. **(A)** EstDZ2 thermostability was evaluated by measurements of residual esterolytic activity after exposure to 45, 50, 55, 60 and 65 °C for up to 24 h. **(B)** EstDZ2 tolerance against various organic solvents was evaluated by measuring its catalytic activity in the presence of 10, 30 and 50% (v/v) of the corresponding solvent. **(C)** EstDZ2 stability against various organic solvents was evaluated by measuring its residual catalytic activity in the presence of 50% (v/v) of the corresponding solvent for a period of 1–3 h. In all panels, the reported values correspond to the mean value from three independent experiments performed in triplicate and the error bars to one standard deviation from the mean value.

When exposed to elevated temperatures for prolonged time periods, EstDZ2 presented moderate stability, as determined by measurements of residual activity. Up to 55 °C, no significant changes in enzymic activity could be detected after exposure up to 24 h (Fig. 3A). When exposed to 60 °C, the enzyme retained more than 50% of its initial activity for at least six hours, while at 65 °C the enzyme was practically inactivated within the first hour of the high-temperature incubation (Fig. 3A).

EstDZ2 was found to be well tolerant against various organic solvents. It exhibited high tolerance to the presence of methanol, ethanol, and acetone, since it retained high levels of catalytic activity when these solvents were added at concentrations as high as 30% (v/v) or more (Fig. 3B). In the presence of acetonitrile and isopropanol, EstDZ2 exhibited intermediate levels of tolerance, while isooctane, n-hexane, and 1-butanol, started to affect its activity at concentrations as low as 10% (v/v). Importantly, EstDZ2 was found to exhibit exquisite stability in the presence of high concentration of organic solvents, as it was found capable of retaining almost full residual catalytic activity after incubation in media containing up to 50% of various solvents for several hours (Fig. 3C).

Subsequently, we went on to test the effects of a range of metal ions, reducing agents and surfactants on the esterolytic efficiency of EstDZ2. We found that the addition of a variety of mono- and divalent metals (Na^+^, K^+^, Ca^2+^, Fe^2+^, Li^2+^, Cu^2+^, Mn^+2^, and Mg^+2^) at 1 mM did not significantly affect EstDZ2 activity (Table 2). Similar effects were observed when the chelating agent ethylenediaminetetraacetic acid (EDTA) was added to the reaction mixture at 1 mM, indicating that the activity of this esterase probably does not depend on a metal cofactor. The presence of nonionic surfactants, such as Tween 20 and Tween 80, did not have a significant impact on EstDZ2 enzymic activity when added at 1% (v/v). On the contrary, 1% Triton X-100 caused significant EstDZ2 inactivation, while the addition of the anionic detergent sodium dodecyl sulfate (SDS) at the same concentration, resulted in complete loss of EstDZ2 activity (Table 2). The addition of 1 mM phenylmethylsulfonyl fluoride (PMSF) resulted in significant inhibition of the enzyme’s esterolytic activity, supporting the *in silico* prediction that EstDZ2 is a serine hydrolase. PMSF inhibits serine hydrolases by covalent linkage to the hydroxyl group of the catalytic Ser residue, a phenomenon that resembles the first transition state in ester hydrolysis, and prevents the formation of the hydrogen-bonding network that is crucial for the catalytic performance of this type of enzymes^4^,^35^.

### Phylogenetic analysis

**Figure 4:**
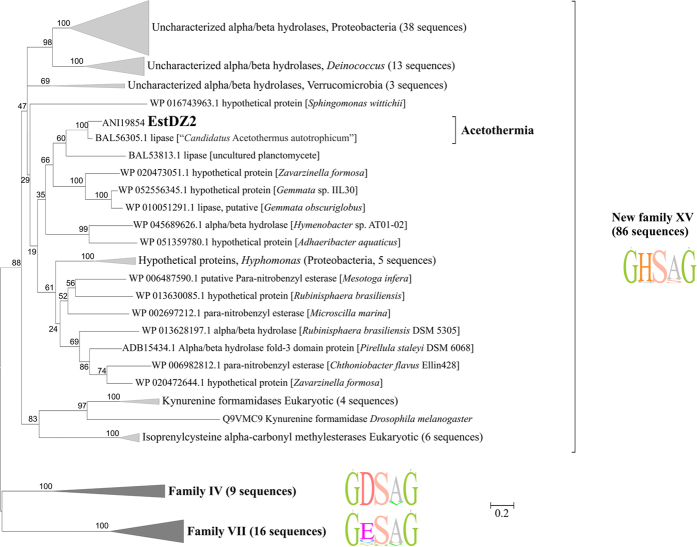
Phylogenetic analysis of EstDZ2 and related sequences. Unrooted Maximum-Likelihood tree of the esterase families XV (this study), IV and VII, which resulted from the phylogenetic analysis described in the Results and Methods sections. The tree with the highest log likelihood is shown. The bootstrap values (100 replicates) are shown next to the branches. The tree is drawn to scale, with branch lengths measured in the number of substitutions per site. Sequence logos indicating the preferred amino acids for each family in the highly conserved catalytic motif GXSXG motif are shown to the right of each family cluster. The tree was generated in MEGA6^84^.

In order to perform phylogenetic analysis of EstDZ2 in a manner that is as unbiased as possible, the Uniprot and Swissprot databases were first searched to identify the protein sequences with the highest degree of sequence similarity to EstDZ2. From this analysis, the top >100 hits corresponding to non-redundant protein sequences with evidence at the protein/transcript levels were selected in order to perform phylogenetic comparison with EstDZ2 (see the Methods section for more details). This analysis revealed that EstDZ2 and its close homologs form a deep branch (Fig. 4). Among the sequences that cluster with EstDZ2, those of bacterial origin have no assigned functions, and correspond to either hypothetical proteins, predicted α/β hydrolases, or sequences with putative carboxyl-esterase/lipase activity. Only a few enzymes within this branch have been characterized functionally, all of which are of plant or animal origin: an isoprenylcysteine methylesterase from *A. thaliana* [UniProtKB/Swiss-Prot: Q94AS5.1]^31^, and two kynurenine formamidases from the fruit fly *Drosophila melanogaster* [UniProtKB/Swiss-Prot: Q9VMC9.1]^36^ and the mouse *Mus musculus* [UniProtKB/Swiss-Prot: Q8K4H1]^37^. The appearance of animal kynurenine formamidases in this cluster is not surprising, as many enzymes of this type are known to resemble carboxyl-esterases more than typical amidases^38^. This similarity is evident both in terms of sequence, since they contain the GXSXG signature catalytic motif of esterolytic enzymes, as well as in terms of folding, as they are known to adopt the typical α/β hydrolase fold, which is characteristic for carboxyl-esterases and lipases^39^. Furthermore, certain kynurenine formamidases have been reported to exhibit esterolytic activity in addition to their ability to hydrolyze amides^38^,^39^.

Strikingly, the nearest homolog of EstDZ2 was an uncharacterized lipase (identity 82%, query coverage 99%) from “*Candidatus* Acetothermus autotrophicum”^40^, an uncultivated representative of *Acetothermia* (OP1) candidate division^41^, which is regarded as one of the deepest bacterial lineages^40^,^42^,^43^. EstDZ2 and this uncharacterized *Acetothermia* lipase are located within a clade of mostly *Planctomycetes* representatives, thus indicating horizontal transfer from *Planctomycetes* to *Acetothermia*. Notably, all five predicted proteins encoded by the genes localized in the same contig as *estDZ2* have their nearest homologs in “*Candidatus* Acetothermus autotrophicum”.

One distinctive feature of EstDZ2 and its homologs is that they share a highly conserved GHSAG catalytic motif. The GHSAG motif is not encountered frequently in any other known family of bacterial esterolytic enzymes^12^,^44^ and its high level of conservation appears to be characteristic for the herein described group of EstDZ2 and its homologous enzymes. Taken together, our results indicate that EstDZ2 belongs to a new family of bacterial esterolytic enzymes, for which we propose the index XV. Based on sequence analyses, enzymes belonging to this new family are most closely related to Family IV esterases (also referred to as hormone-sensitive lipases, HSLs) and –to a smaller extent– to Family VII esterases, but they are sufficiently distinct in terms of sequence characteristics. The most notable among these differences is the presence of a dominant GHSAG catalytic motif, whereas Family IV and Family VII esterases are characterized by the presence of dominant GDSAG and GESAG motifs, respectively (Fig. 4; Table 3).

### Structural model of EstDZ2

**Figure 5:**
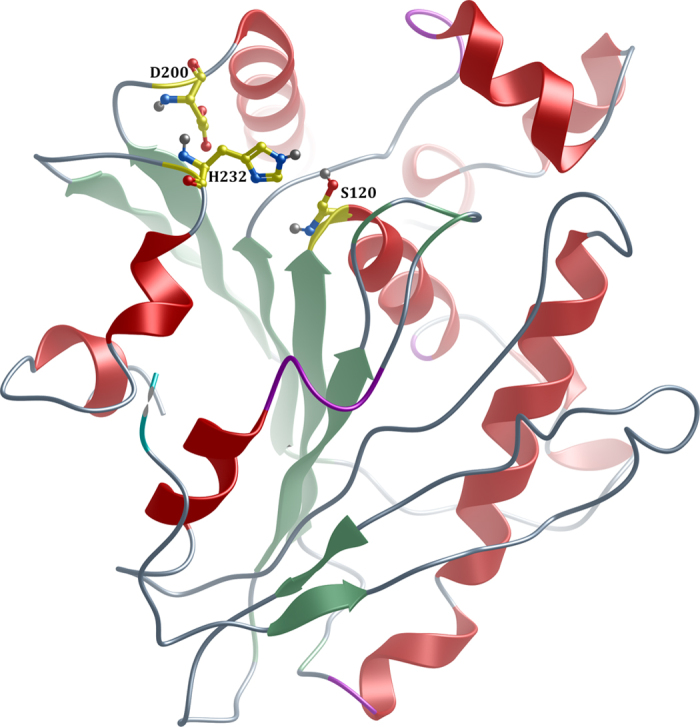
The modelled three-dimensional structure of EstDZ2. Residues Ser120, Asp200 and His232 forming the catalytic triad are indicated in ball-and-stick representation. The figure was prepared using *MolSoft*^86^.

Modelling studies to predict the three-dimensional structure of EstDZ2 were performed by selected automated web-servers for protein structure homology modelling included in “Protein Model Portal”^45^, such as SWISS-MODEL^46^, M4T^47^, InFOLD2^48^, Phyre2^49^ and I-TASSER^50^, by applying standard protocols. Comparison of the overall structures generated by the different models, showed that the architecture of the catalytic domain was highly conserved, while differences were mainly located in loop regions (data not shown). Therefore, we focused on the results produced by the Iterative Threading ASSEmbly Refinement program (I-TASSER)^50^, since it is the most recently developed suite that utilizes iterative threading assembly simulations, coupled with secondary structure enhanced Profile-Profile threading alignment and *ab initio* Monte Carlo simulations for unaligned regions. The top ten threading templates selected by I-TASSER included microbial esterases and the *D. melanogaster* kynurenine formamidase, with sequence identity ranging from 34 to 23% (alignment coverage 96–92%). This finding is in perfect agreement with our phylogenetic analysis, which classified EstDZ2 among putative esterases and indicated that the fruit fly kynurenine formamidase is the most closely related protein sequence of known structure to EstDZ2 (Fig. 4). The final predicted model structure of EstDZ2 produced by I-TASSER was selected according to the confidence and structural similarity scores (see Methods for details) and is shown in Fig. 5.

**Figure 6:**
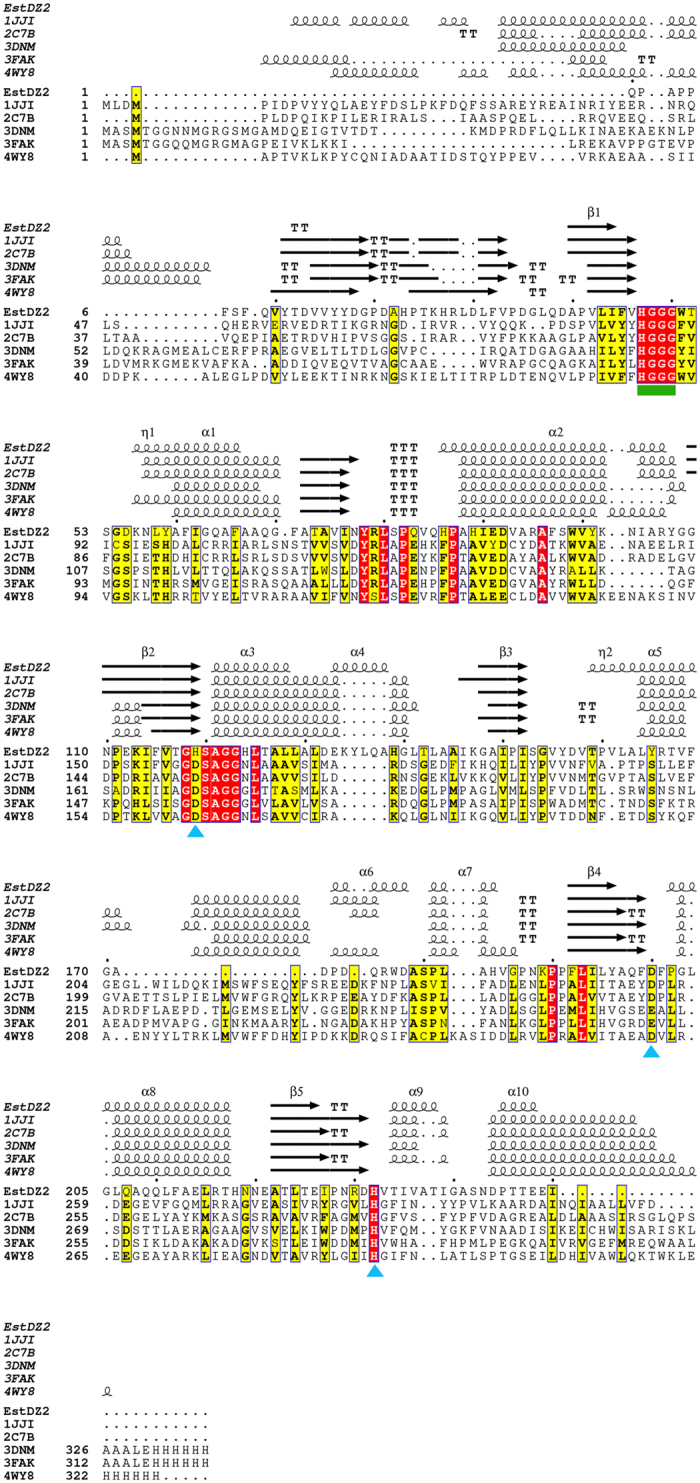
Multiple sequence alignment of EstDZ2 and selected Family IV esterases of known 3D structure. The absolutely conserved amino acids are highlighted in red, while similar ones in yellow. The conserved tetrapeptide motif HGGG (residues 47–50) is indicated by a green box, whereas the catalytic site residues (Ser120, Asp200, His232) by blue triangles. Elements of secondary structure for all proteins are denoted as α (α helix), β (β sheet), η (random coil), and Τ (β turn). Sequence alignment was performed using Clustal Omega^87^ and ESPript^88^.

The predicted EstDZ2 structure is characterized by the α/β hydrolase fold, which is typical for esterolytic enzymes, forming a twisted β-sheet, sandwiched between two layers of helices (Fig. 5). Residues Ser120, Asp200 and His232 are predicted to constitute the catalytic triad. These residues are found to be in close proximity in the model structure, ready to participate in catalysis. The position of their backbone atoms was found to be absolutely conserved in all model structures derived with other utilized structure prediction tools (data not shown). Ser120 is part of the highly conserved GXSXG catalytic motif^30^, as also demonstrated in the sequence alignment of EstDZ2 and other members of the closely related Family IV esterases with known 3D structure (Fig. 6). The participation of Ser120 to the catalytic mechanism of EstDZ2 was verified by mutational analysis, as the amino acid substitution S120A abolished esterolytic activity (Fig. 1B). The tetrapeptide sequence HGGG (residues 54–57 in EstDZ2), which contributes to the formation of the oxyanion hole^30^ is also absolutely conserved (Fig. 6). Inactivation of the enzyme’s esterolytic activity by the serine-specific inhibitor PMSF^51^ (Table 2) supports further the prediction that EstDZ2 is a serine hydrolase.

**Figure 7:**
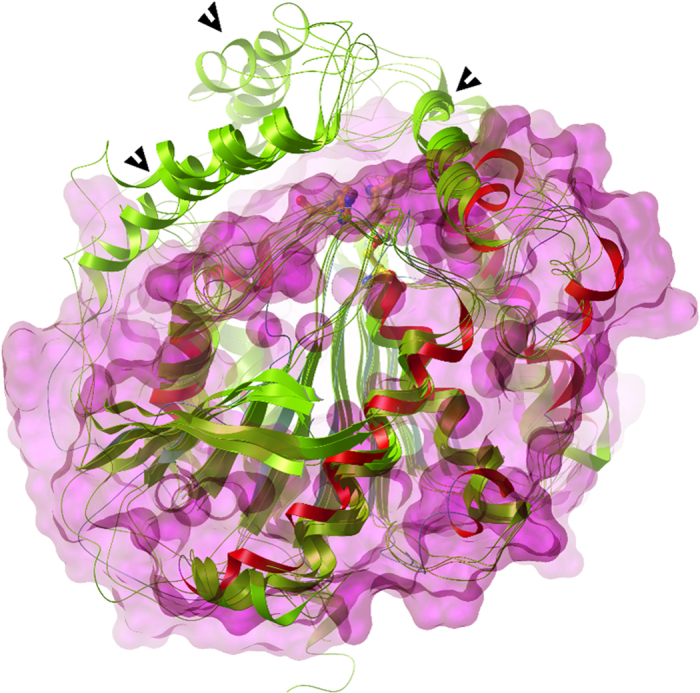
Superposition of the modelled EstDZ2 structure onto the structures of five representative Family IV esterases. The modelled EstDZ2 structure is shown in red, while the structures of the five representative Family IV esterases described in Fig. 6 are shown in green. The molecular surface of EstDZ2 is depicted in magenta and demonstrates that the conserved “cap” domain for Family IV esterases (denoted by black arrows) formed by the N-terminal helices is absent from EstDZ2, while only part of the C-terminal helices contributing to the formation of the “cap” domain is present.

In order to perform a structural comparison of EstDZ2 with closely related bacterial esterolytic enzymes, we superimposed the modelled EstDZ2 structure with five representative carboxyl esterases of known structure, which have been classified in the literature as Family IV members, the most closely related family to EstDZ2 and other Family XV enzymes. In accordance with conclusions from previous studies, all five Family IV enzymes were characterized by the presence of multiple N- and C-terminal residues belonging to structurally conserved α-helices of various lengths that form an extended “cap” domain (Fig. 7), a structural feature which is highly conserved among members of this family^30^,^52^,^53^. This domain acts as a shield for the catalytic site of many esterolytic enzymes and appears to play a key role in several functional aspects of Family IV esterases, such as activity, substrate specificity, and thermostability^52^. EstDZ2, on the other hand, seems to completely lack these N-terminal helices, while only part of the C-terminal ones are present, resulting in a structure which provides a different type of shielding of the EstDZ2 active site compared to Family IV enzymes (Figs 6 and 7). This structural difference in the environment of the catalytic site is illustrated by the superposition of the EstDZ2 predicted structure on an ensemble of representative Family IV esterases (Fig. 7), and indicates that EstDZ2 may be distinct from related enzymes not only in terms of sequence, but also in terms of structure.

## Discussion

In this study, we have identified a new thermostable carboxyl esterase by subjecting the metagenomic DNA isolated from a hot spring located in Kamchatka-Russia, to bioinformatic screening for new lipolytic enzymes. The identified esterase, termed EstDZ2, was cloned, purified from *E. coli* and characterized biochemically. While the physiological role of this new enzyme is unknown, EstDZ2 presented a typical esterolytic profile and was found to be highly active against tributyrin and pNP-esters of fatty acids of medium chain length. For pNP-butyrate (C4), the catalytic efficiency (*k*_cat_/*K*_m_) of EstDZ2-mediated hydrolysis was found to be 14,375 s^−1^mM^−1^. Compared to esterolytic enzymes deposited in the BRENDA database^54^, a collection of enzyme and metabolic information based on primary literature, this level of catalytic efficiency is markedly higher than that of most characterized esterases. Out of the twenty esterases in the database that have been assayed against pNP-butyrate, sixteen exhibit a catalytic efficiency that is one or two orders of magnitude lower than that of EstDZ2. On the contrary, comparison with the rest of the four esterases, which are present in this group and have been found to exhibit higher catalytic efficiency, EstDZ2 exhibits a *k*_cat_/*K*_m_ value that is only two- to three-fold lower. Among this set, the enzyme with the closest catalytic efficiency value for pNP-butyrare *(k*_cat_/*K*_m_ = 10,000 s^−1^mM^−1^) is the esterase Est from the hyperthermophilic archaeon *Pyrobaculum calidifontis*^55^.

EstDZ2 was found to be a moderately thermostable enzyme, with a half-life of more than six hours at 60 °C. Importantly, it exhibited good tolerance and exquisite stability when challenged against high concentrations of organic solvents. Compared to the recently discovered esterase EstOF4^56^, EstDZ2 demonstrated greater tolerance to the presence of all organic solvents tested. When compared in terms of organic solvent stability with other recently discovered thermostable and biotechnologically promising esterolytic enzymes, such the esterases Pf_Est from *Pyrococcus furiosus*^57^ and EstLiu from the marine bacterium *Zunongwangia profunda*^16^, EstDZ2 exhibited superior stability against commonly utilized solvents, such as methanol, ethanol and isopropanol. In particular, while EstDZ2 activity remained practically unaffected in media containing 50% of these solvents after incubation for up to 3 h, exposure of Pf_Est to the same conditions resulted in loss of half of its maximal activity only after 30 min of incubation^57^. Similarly, when EstLiu was incubated under similar conditions, 40–80% of its maximal activity was lost within the first 40 min of solvent exposure^16^.

When compared to other known industrially relevant enzymes, the biochemical profile of EstDZ2 resembles that of the landmark lipolytic enzyme Novozym 435, as described in the BRENDA database^54^. Similarly to EstDZ2, Novozym 435 exhibits optimal activity at 50 °C and functions in the temperature range of 10–70 °C and at pH values between 7 and 9. Furthermore, tributyrin has been reported to be the natural substrate of Novozym 435, a preferred substrate also for EstDZ2. Finally, EstDZ2 retains high levels of enzymic activity in media containing organic solvents, a characteristic which is also shared with Novozym 435, and which has been exploited several times for performing biocatalytic reactions in non-aqueous media^58^,^59^,^60^.

Phylogenetic analysis indicated that EstDZ2 belongs to a putative new family of bacterial esterolytic enzymes, for which we have proposed the index XV. The amino acid sequence of EstDZ2 did not cluster with that of any previously characterized bacterial esterolytic enzyme. Furthermore, EstDZ2 and EstDZ2-resembling sequences were characterized by a distinctive GHSAG pentapeptide catalytic motif, which is not frequently encountered in any described family of bacterial esterases/lipases. In the most closely related Family IV enzymes, the His residue of the GHSAG motif in EstDZ2 and homologous enzymes, is replaced by an Asp residue, yielding the characteristic GDSAG pentapeptide catalytic motif for this family of esterolytic enzymes^12^.

Multiple sequence alignment, coupled with homology modelling for the construction of a three-dimensional structure of the new enzyme, supported further the notion that EstDZ2 belongs to a new family of bacterial esterolytic enzymes. EstDZ2 seems to lack a significant part of the so-called “cap” domain, whose extended structure is characteristic for Family IV esterases^30^,^52^,^61^,^62^. In accordance with the above prediction, EstDZ2 activity was found to be inhibited by PMSF. Esterases with a full-size “cap” domain are typically found to be unaffected by the addition of this serine hydrolysis-inactivating agent, as this domain acts as a “shield” over the active site and protects the catalytic Ser^66^,^67^,^68^. Other known esterolytic enzymes with minimal “cap” domains include a *Streptomyces exfoliate*s lipase^63^, and two carboxyl esterases from *Lactobacillus plantarum*^64^ and *Thermogutta terrifontis*^65^. Determination of the EstDZ2 3D structure will allow a direct comparison to Family IV enzymes and other esterase families, thus providing insights on the mechanism of action of EstDZ2, both in its native form and in complex with substrate analogues. EstDZ2 structural studies using X-ray crystallography are currently underway in our laboratories.

According to our phylogenetic analysis, horizontal gene transfer was likely the main route of evolution of the representatives of the XV family of esterases and their homologues among eukaryotes and various bacterial phyla, including the deepest lineages, such as the *Acetothermia* (OP1) candidate division. Analysis of the “*Candidatus* Acetothermus autotrophicum” draft genome (four contigs) characterized its putative physiology as facultative anaerobic, acetogenic, chemolithoautotrophic or chemoorganoheterotrophic. Although the natural substrates of EstDZ2 are currently unknown, our findings provide support to the claim made previously by Takami *et al*.^40^ for a possible heterotrophic lifestyle of *Acetothermia* representatives, and indicate that the spectrum of their possible substrates may include esters and lipids. The discovery of EstDZ2 suggests that OP1 may play an important role in the hydrolysis of lipids, providing more easily utilized substances, such as glycerol, fatty acids, and/or the products of their degradation to other members of the microbial consortium.

The ability of EstDZ2 to efficiently catalyze the hydrolysis of short to medium-length acyl chains, combined with its good thermostability and optimum activity at moderately high temperatures (50–60 °C), suggest that EstDZ2 could be utilized in food-processing applications, such as dairy product modifications and synthesis of flavor compounds. Its tolerance against non-ionic surfactants and metal ions renders EstDZ2 a candidate enzyme for use in detergent mixtures and emulsifiers, while its good tolerance and excellent stability against organic solvents, could signify its potential use towards the production of biodiesel and biopolymers^5^,^19^,^69^.

In conclusion, EstDZ2 constitutes a new candidate enzyme for industrial biotransformations, with novel sequence characteristics that appear to define a new family of esterolytic enzymes.

## Methods

### Reagents and chemicals

All chemical reagents used in this study were purchased from Sigma-Aldrich (St. Louis, USA). All molecular biology-related products (restriction enzymes, protein markers, etc.) were purchased from New England BioLabs (Ipswich, USA).

### Environmental sampling

A detailed description of the environmental sampling site and DNA extraction procedures is given by Menzel *et al*.^28^. Briefly, 150 ml of wet mud sample was collected and processed on-site to fix the total DNA in RNAlater Stabilization Reagent (Qiagen - Hilden, Germany) before further processing in the lab. The microbial cells contained in the collected sample were disrupted by freeze-thaw cycles, lysozyme, and sodium lauroyl sarcosinate treatments. Total DNA was extracted by a standard phenol extraction method and purified from residual polysaccharides and humic substances. The final preparation contained 1.8 μg of metagenomic DNA as determined by dsDNA BR assay on a Qubit 2.0 Fluorometer (Invitrogen - Waltham, USA) according to the manufacturer’s instructions.

### Bioinformatic analyses

The isolated metagenomic DNA was sequenced on a Roche/454 Titanium FLX next-generation sequencing platform and assembled into contigs using three different assembly tools as described by Menzel *et al*.^28^. For the *de novo* prediction of coding sequences in the generated contigs, three different types of software were utilized, each based on a different machine-learning model: MetaGeneAnnotator^70^, MetaGeneMark^71^ and Prodigal^72^. The combined results of all three analyses consisted of 32,525 putative gene sequences. These were subsequently submitted to a similarity search using BLASTp and DELTA-BLAST^73^ against sequences with lipolytic activity deposited in both NCBI-nr and UniProt/Swiss-Prot^74^. The generated hits were imported into a local MySQL^75^ database by running an in-house Python script, and comparative tables were created using appropriate search queries. These tables comprised the highest identity scoring results from both databases for every single sequence, including the corresponding EC numbers from the Uniprot/Swiss-Prot database. The sequences were also examined for Pfam domains using HMMER (Hmmscan tool)^76^ against the Pfam-A database. The generated results from the Hmmscan analysis were also imported in a local MySQL database via another in-house Python script and were queried in order to return sequences with domains related to esterolytic activity. From the BLAST hits, the ones with the highest similarity score to sequences with esterolytic activity (EC number: 3.1.1.x) were filtered out from the UniProt/Swiss-Prot database and were compared with the corresponding hits from the NCBI-nr database. The sequence subsequently nominated as EstDZ2α was one of the hits considered of high interest as a candidate esterolytic enzyme, since HMMscan analysis returned a statistically significant Pfam-A match from the list of lipolytic-related domains, an α/β hydrolase_3 domain (PF07859). Further curation of the sequence included putative EC assignment by exploiting a machine-learning based tool; EFICAZ 2.5^77^ returned a low-confidence (EFICAz_components: CHIEFc_FDR, MTTSI_bin: 2) EC prediction of 3.1.1.x. Furthermore, EstDZ2α showed 23% amino acid sequence identity (e-value 6 × 10^−24^, query coverage 90%, positive percentage 42%) with a previously characterized isoprenylcysteine alpha-carbonyl methylesterase^31^ in UniProt (Accession number: Q94AS5.1). The corresponding hit in NCBI-nr had 82% identity (e-value < 10^−161^, query coverage 99%, positive percentage 88%) with a sequence annotated as a putative lipase from an uncultured *Acetothermia* bacterium (Accession number: BAL56305.1).

### Plasmid construction

pET-EstDZ2α was constructed by amplifying *estDZ2a* from the isolated metagenomic DNA by PCR using primers containing XbaI (5′- AAAAATCTAGAAGGAGGAAACGATGCGCACAATACTCTATGCAACTCTAGC-3′) and HindIIΙ (5′- AAAAAAAGCTTTTAAATCTCTTCGGTCGTGGGATCATTAGACGCTCCAATGGTGGCG -3′) restriction sites (underlined) and was cloned into the expression vector pET-28a(+) (Novagen - Darmstadt, Germany). pET-EstDZ2 was constructed by replacing the sequence encoding for amino acids 2–21 of EstDZ2α with a hexa-histidine tag. For this, *estDZ2* was amplified from pET-EstDZ2α using the forward primer 5′- AAAAATCTAGAAGGAGGAAACGATGCACCACCACCACCACCACCACCACGCGCCGCCGTTCTCGTTCC -3′ (XbaI restriction site underlined and the hexa-histidine tag doubly underlined) and the reverse primer utilized for the cloning of *estDZ2a*. For the generation of the EstDZ2 variant S120A, overlap extension PCR was carried out using the forward primer (5′-GAAGATCTTCGTGACGGGGCACgCAGCTGGGGGACATCTGACGGCT-3′) and the reverse primer (5′-AGCCGTCAGATGTCCCCCAGCTGcGTGCCCCGTCACGAAGATCTTC-3′), in combination with the aforementioned external primers. The mutant gene was cloned into pET-28a(+) using the same cloning strategy as described above, thus leading to the generation of the recombinant plasmid pET-EstDZ2α(S120A). The correct sequence of all constructs was verified by standard DNA sequencing.

### Protein expression and purification

BL21 (DE3) cells carrying the pET-EstDZ2 plasmid were grown in LB broth containing 50 μg/ml kanamycin at 37 °C under constant shaking until the culture reached an OD600 of about 0.5. At that point, the expression of *estDZ2* was induced by the addition of 0.2 mM IPTG followed by overnight incubation at 25 °C with shaking. For EstDZ2 purification, the cells from a 500 mL culture grown in a 2 L shake flask were harvested, washed, re-suspended in 10 mL equilibration buffer NPI20 enhanced with 1% Triton X-100 (v/v), and lysed by brief sonication steps on ice. The cell extract was clarified by centrifugation at 10,000×g for 15 min at 4 °C and the supernatant was incubated for 1 h at 60 °C in order to denature *E. coli* proteins. After this heat-treatment step, the precipitated material was removed again by centrifugation at 10,000 × g for 15 min at 4 °C. The supernatant was collected and mixed with 0.5 mL Ni-NTA agarose beads (Qiagen - Hilden, Germany) and shaken mildly for 1 h at 4 °C. The mixture was then loaded onto a 5 mL polypropylene column (Thermo Fisher Scientific - Waltham, USA), the flow-through was discarded, and the column was washed with 10 mL of NPI20 wash buffer containing 1% (v/v) Triton X-100. Next, Triton X-100 was washed away by passing 20 mL of standard NPI20 wash buffer. EstDZ2 was eluted using 1 mL of NPI200 elution buffer. All buffers used for purification were prepared according to the manufacturer’s protocol (Qiagen - Hilden, Germany) unless stated otherwise. Imidazole was subsequently removed from this protein preparation using a Sephadex G-25 M PD10 column (GE Healthcare - Chicago, USA). Protein concentration was estimated according to the assay described by Bradford^78^ using bovine serum albumin as a standard. The purified protein was visualized by SDS-PAGE analysis and staining with Coomassie blue or Western blotting using an anti-polyhistidine monoclonal antibody conjugated with horseradish peroxidase (Sigma - St. Louis, USA), and stored at -80 °C after the addition of 50% (v/v) glycerol.

### Protein electrophoresis and zymography

*E. coli* BL21(DE3) cells were transformed with either pET-28a(+) or pET-EstDZ2α, grown in LB medium at 37 °C with shaking until the culture reached an OD 600 nm of 0.5, at which point 0.2 mM IPTG was added to induce *estDZ2α* overexpression. After additional incubation at 25 °C for 12 h, the cells were lysed by brief sonication and the proteins contained in 15 μl of total cell lysate were separated by native PAGE. To avoid denaturation, protein separation was performed at 120 V/35 mA for two gels, and the tank was immerged in ice-cold water. Esterase activity was assayed by zymography according to Dherbecourt *et al*.^79^ with minor modifications: the gel was rinsed in distilled water and incubated for 30 min at 40 °C in 0.1% Fast Red TR-salt (4-chloro-2-methylbenzenediazonium salt) in 0.1 M Tris-HCl buffer (pH 7.0) containing 2% of a 1% (v/v) 1-naphthyl acetate solution in acetone. Esterolytic activity was visualized by the appearance of a band of red-brown color.

### Enzyme activity assays

For the initial specificity analysis using natural substrates, cell lysates were assayed against ethyl-acetate, butyl-acetate, cinnamyl-acetate, tributyrin and triolein. The substrates were used at a 5 mM concentration in 100 μL reactions, containing a 25 mM Tris-HCl pH 7.3 buffer with 0.01% bromothymol blue (BTB) as a pH indicator. The reaction was carried out for 15 min under constant agitation. Natural oils, such as olive oil, sunflower oil and palm oil, were tested in the same reaction at 1% (v/v). For biochemical characterization, the catalytic activity of EstDZ2 was determined by quantification of the amount of *p*-nitrophenol released from the substrate by photometric measurement at 410 nm. Unless stated otherwise, the 100 μL standard reaction mixture consisted of 25 mM Tris-HCl pH 8 buffer with 0.05% Triton X-100 (v/v), 2 mM pNP-butyrate and 1 μg of pure enzyme, and was carried out for 10 min at 50 °C on a MJ Research thermal cycler, with a pre-incubation setting of the buffer to the target temperature, before the enzyme was added. Enzymic activity was recorded using a Safire II-Basic plate reader (Tecan, Austria) by measuring the absorbance of the released pNP at 410 nm, immediately after the reaction was completed. For the substrate specificity experiments, a range of different pNP-fatty acyl esters, such as acetate (C2), butyrate (C4), octanoate (C8), decanonate (C10), laurate (C12), and palmitate (C16) were used at concentrations ranging from 0.1 to 1 mM. For the determination of the enzyme’s optimal pH, reactions were carried out at 40 °C in 25 mM acetate, phosphate, Tris-HCl and glycine-NaOH buffers for pH values 4–6, 6–7, 7–9 and 9–10, respectively. Activity was measured by recording absorbance at 348 nm, the isosbestic point of pNP, so as to exclude the pH effect on the readings, as described previously^80^. Temperature profiling of EstDZ2 was performed by incubating the standard reaction at temperatures ranging from 25 to 70 °C. The assays for the determination of EstDZ2 tolerance to the presence of metal ions, detergents and organic solvents were also executed in the standard reaction with the only difference being the addition of these agents at the specified concentrations. The standard reaction without addition of the tested denaturant was recorded as maximal (100%) activity, while reactions that included the tested denaturing agents but no enzyme, were used as blanks. Organic solvent stability was evaluated after incubating the enzyme in 50% of the tested solvents for time periods of 1, 2 and 3 h. Subsequently, the incubation medium was diluted to remove the solvent, and the residual activity of EstDZ2 was measured in the standard reaction. All measurements were obtained from at least three independent experiments carried out in triplicates.

### Phylogenetic analysis

The analysis dataset included the amino acid sequence of EstDZ2α, along with its 100 top BLAST hits obtained via the NCBI BLASTp utility (http://blast.ncbi.nlm.nih.gov/Blast.cgi?PAGE=Proteins; threshold: 0.01; word length: 2; non-redundant protein sequences (nr) database), and the 31 top BLAST hits, obtained via the Uniprot BLASTp utility (http://www.uniprot.org/blast/; threshold: 0.01; UniProtKB-SwissProt database). For the latter, only complete proteins with evidence at the protein/transcript levels were retrieved. The results were filtered through an 0.95% identity filter using the CD-hit utility (http://weizhong-lab.ucsd.edu/cdhit_suite/cgi-bin/index.cgi?cmd=h-cd-hit)^81^ to eliminate duplicates and reduce redundancy. Preliminary investigations revealed that EstDZ2α is mostly similar to Family IV and VII esterases. Based on this, three additional Family IV (Uniprot accession numbers: O52270, Q8NKS0, A0A068LG40) and three additional Family VII (P37967, Q8GCC7, Q9Z545) proteins were included in the analysis. The final dataset consisted of 111 sequences. The dataset was aligned in Geneious 8.1 using MAFFT v.7 (algorithm E-INS-i, default parameters)^82^. The evolutionary history was inferred by using the Maximum Likelihood method based on the Whelan And Goldman + Freq. model^83^. All positions with less than 95% site coverage were eliminated. There were a total of 196 positions in the final dataset. The tree was generated in MEGA6^84^ and the tree with the highest log likelihood (-30586.7910) was selected. Sequence logos were constructed with Geneious 8.1.

### Modelling studies of EstDZ2

Modelling studies on the three-dimensional structure of EstDZ2 were performed by employing a variety of automated web-servers for protein structure homology modelling and applying standard protocols as implemented in “Protein Model Portal”^45^. These include SWISS-MODEL^46^, M4T^47^, InFOLD2^48^, Phyre2^49^ and I-TASSER^50^. Model structures of EstDZ2, were created based on templates derived from previously determined structures that exhibited esterolytic and/or other activities, i.e. an engineered feruloyl esterase derived from a soil metagenomic library (PDB code 4ZRS), a putative thioesterase from *Silicibacter* sp. tm1040 (PDB code 2PBL), esterase B from *Lactobacillus rhamnosis* (PDB code 4N5I), the Ttest2 esterase from *Thermogutta terrifontis* (PDB code 5AO9), a kynurenine formamidase from *D. melanogaster (*PDB code 4E11), and a carboxyl esterase from *Ferroplasma* (PDB code 3WJ1). From the top five final models predicted by I-TASSER, the most recently developed suite, only one was created with reliable prediction scores (C-score 0.61 and TM-score 0.8 ± 0.09). The C-score represents the confidence of the generated model, with values ranging between zero and one, while the TM-score is a measure of the global structural similarity between the predicted EstDZ2 structure and that of template proteins. Superposition of the modelled structure with Family IV representatives was performed by COOT^85^ using secondary structure elements. Visual representation of the modelled structure was performed with the molecular graphics software *MolSoft*^86^.

## Additional Information

**Accession codes:** The nucleotide sequence of *estDZ2a* has been deposited in GenBank under accession code KX301277.

**How to cite this article**: Zarafeta, D. *et al*. Metagenomic mining for thermostable esterolytic enzymes uncovers a new family of bacterial esterases. *Sci. Rep.* **6**, 38886; doi: 10.1038/srep38886 (2016).

**Publisher's note:** Springer Nature remains neutral with regard to jurisdictional claims in published maps and institutional affiliations.

## Supplementary Material

Supplementary Information

## Figures and Tables

**Table 1:** Kinetic parameters of EstDZ2 hydrolysis of various pNP-esters.

Substrate (pNP ester)	*K*_m_ (mM)	*V*_max_ (μmol∙min^−1^∙mg^−1^)	*k*_cat_ (s^−1^)	*k*_cat_/*K*_m_ (s^−1^mM^−1^)
Acetate (C2)	0.67 ± 0.15	36.8 ± 5.2	1,226	1,757
Butyrate (C4)	0.32 ± 0.09	139.6 ± 13.3	4,653	14,375
Caprylate (C8)	0.20 ± 0.02	61.8 ± 3.3	2,060	10,437
Caprate (C10)	0.12 ± 0.01	28.1 ± 0.5	936	7,551

**Table 2:** Effect of metal ions, surfactants and other denaturing agents on EstDZ2 activity.

Metal ion or chemical agent	Concentration	Relative activity (%)
None	—	100.0 ± 7.8
K^+^	1 mM	102.5 ± 9.2
Mn^2+^	1 mM	106.7 ± 5.0
Ca^2+^	1 mM	93.9 ± 5.4
Zn^2+^	1 mM	87.8 ± 1.3
Li^2+^	1 mM	112.9 ± 9.2
Mg^2+^	1 mM	104.6 ± 4.0
Na^+^	1 mM	99.3 ± 5.9
Fe^3+^	1 mM	95.9 ± 9.6
Cu^2+^	1 mM	109.8 ± 8.8
EDTA	1 mM	113.4 ± 7.0
PMSF	1 mM	31.0 ± 5.6
Triton X-100	1% (v/v)	55.0 ± 8.0
Tween 20	1% (v/v)	104.1 ± 9.1
Tween 80	1% (v/v)	95.9 ± 5.3
SDS	1% (w/v)	Undetectable

**Table 3:** List of the amino acid sequences corresponding to the highly conserved oligopeptide GXSXG catalytic motif that appears as the most dominant one in the previously described bacterial esterase families following the Arpingy and Jaeger classification^5^ and numbering (Families I-XIV)^12^ and of additional families of bacterial esterolytic enzymes that do not follow the Arpingy and Jaeger numbering.

Family of bacterial esterolytic enzyme	Conserved catalytic motif
Family I	GHSQG
Family II	GDSL
Family III	GXSMG
Family IV	GDSAGG
Family V	GXSMGG
Family VI	GFSQG
Family VII	GESAG
Family VIII	GGSVG
PhaZ7 (Family IX)^89^	AHSMG
EstD (Family X)^8^	GHSLG
LipG (Family XI)^9^	GHSLGG
LipEH166 (Family XII)^10^	GHSLG
Est30 (Family XIII)^11^	GLSLGG
EstA3 (Family XIV)^90^	CHSMG
EstA^91^	GHSMG
VLip509^92^	GHSLGG
EstD2^15^	GHSQG
EstZ3, EstGK1^93^	XHSQX
FLS18^94^	AHGMG
EM3L4^95^	GHSQG
EstWSD^14^	GHSQG
EstLiu^16^	GFSAG
Family XV (this study)	GHSAG

**Note:** The list of additional families of bacterial esterolytic enzymes that do not follow the Arpingy and Jaeger numbering is indicative and may not be exhaustive.
