# Supplementary material for: Metagenomic mining for thermostable esterolytic enzymes uncovers a new family of bacterial esterases
Source: Sci Rep. 2016 Dec 19;6:38886. doi: 10.1038/srep38886 (PMC5171882; doi:10.1038/srep38886)
Supplement: Supplementary Information [file srep38886-s1.pdf]

Supplementary Information

Metagenomic mining for thermostable esterolytic enzymes uncovers a new family of bacterial esterases

Dimitra Zarafeta[1,2], Danai Moschidi[2], Efthymios Ladoukakis[2], Sergey Gavrilov[3], Evangelia D. Chrysina[1], Aristotelis Chatziioannou[1], Ilya Kublanov[3,] Georgios Skretas[1*], Fragiskos N. Kolisis[2*]

[1]*Institute of Biology, Medicinal Chemistry & Biotechnology, National Hellenic Research Foundation, Athens, Greece*

[2]*Laboratory of Biotechnology, School of Chemical Engineering, National Technical University of Athens, Athens, Greece*

[3]*Winogradsky Institute of Microbiology, Research Center for Biotechnology Russian Academy of Sciences, Moscow, Russian Federation*

*Correspondence should be addressed to:

Georgios Skretas: gskretas@eie.gr or

Fragiskos N. Kolisis: kolisis@chemeng.ntua.gr

Supplementary Figures

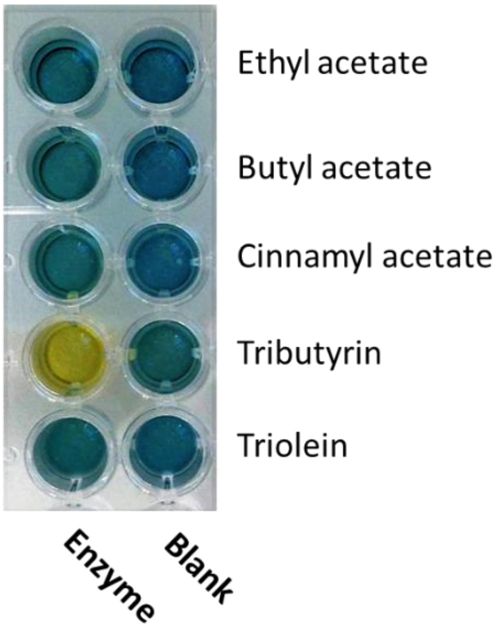

Supplementary Fig. S1. EstDZ2 esterolytic activity against natural substrates. Clarified lysates of *E. coli* BL21(DE3) cells in the presence (Enzyme) and absence (Blank) of *estDZ2a* overexpression were assayed against natural substrates of estrolytic enzymes (5 mM in Tris-HCl, pH 7.3) in reactions containing 0.01% bromothymol blue as a pH indicator.

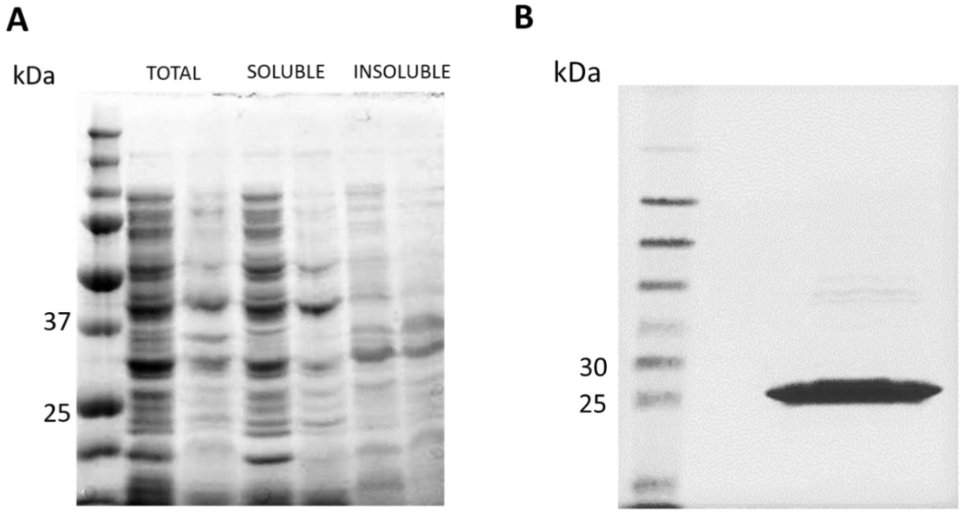

Supplementary Fig. S2. EstDZ2 purification. (A) SDS-PAGE analysis of *E. coli* BL21(DE3) cell lysates overexpressing *estDZ2* (+) and cells carrying an empty vector (-). **(B)** SDS-PAGE analysis of isolated EstDZ2 after IMAC purification and staining with Coomassie blue. Molecular weight markers are indicated on the left hand side of each gel.

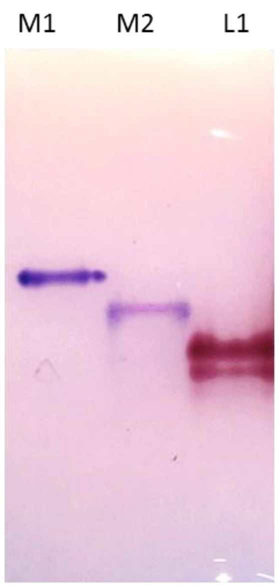

Supplementary Fig. S3. Native PAGE analysis of purified EstDZ2. Fast Red staining using 1-naphthyl acetate as a substrate followed by staining with Coomasie blue. **M1**: Protein marker containing bovine serum albumin (BSA) (MW: 66.5 kDa); **M2**: Protein marker containing human Cu/Zn superoxide dismutase 1 (SOD1) (MW: 32.5 kDa); **L1**: Purified EstDZ2 (predicted MW: 28.4 kDa).

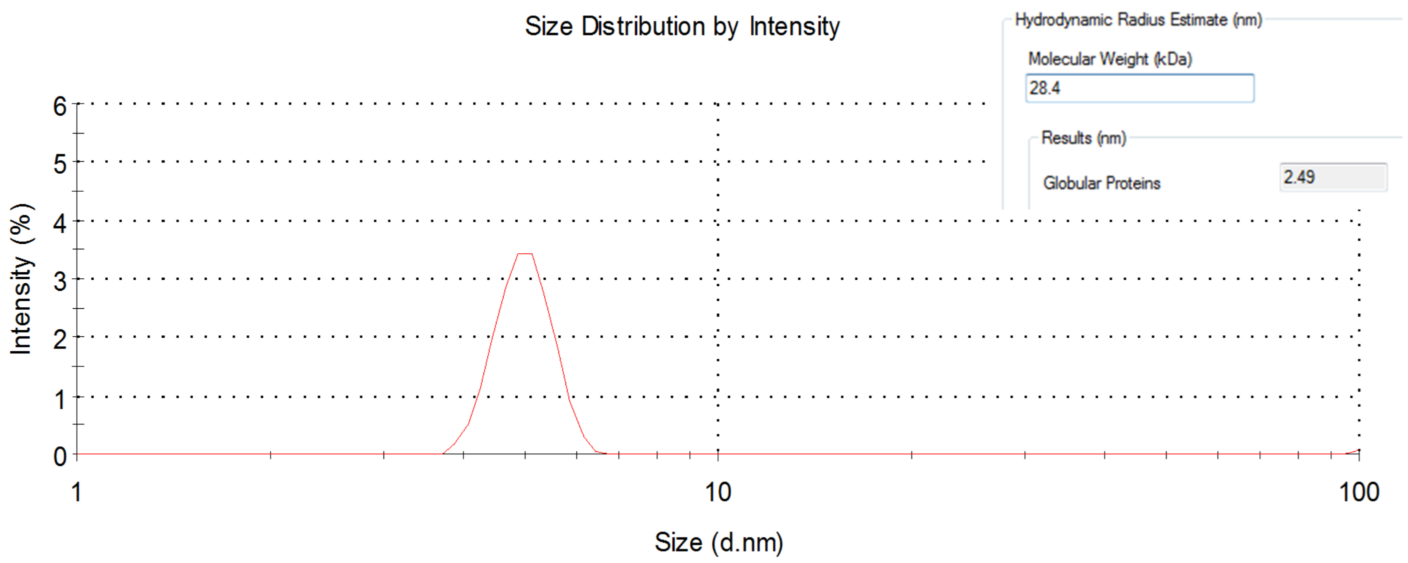

Supplementary Fig. S4. Dynamic light scattering (DLS) analysis of purified EstDZ2. The diameter of the particle size detected was measured at 4.98 nm (2.5 nm radius). The estimated molecular mass for a globular protein of this radius is 28.7 kDa, which is in perfect agreement with the predicted molecular mass for monomeric EstDZ2 (28.4. kDa). DLS analysis was carried out used was a Zetasizer Nano ZS (Malvern Instruments, UK) equipped with a He-Ne laser (632.8 nm) using a non-invasive back scatter (NIBS) technology. Analysis conditions: 25 ^{o}C, protein concentration: 0.45 mg/mL in Tris-HCl 50 mM pH 8 buffer.

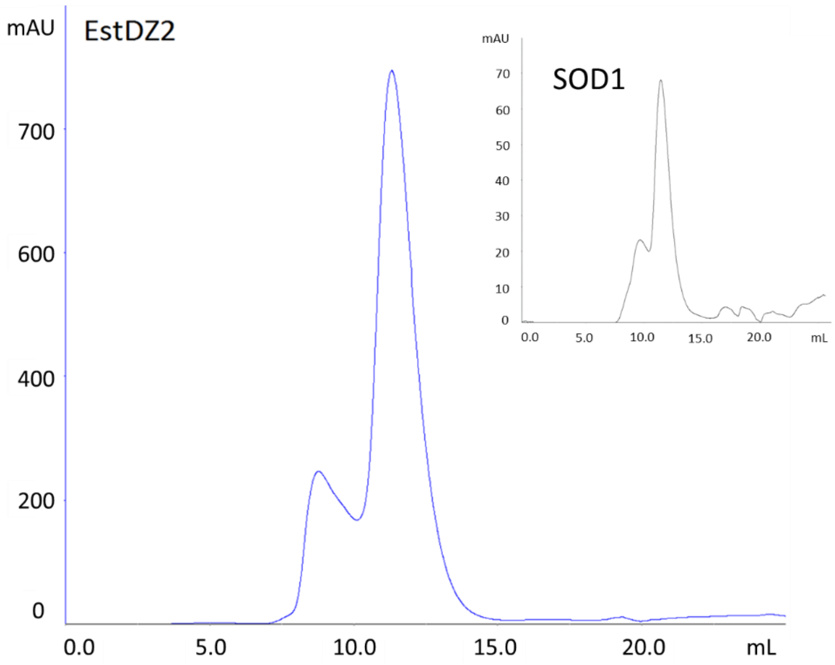

Supplementary Fig. S5. Size exclusion chromatography analysis of purified EstDZ2. Purified dimeric human Cu/Zn superoxide dismutase 1 (SOD1) (32.5 kDa) was used as a standard to evaluate the native molecular weight of EstDZ2. EstDZ2 (predicted MW 28.4 kDa) eluted from a GE Superdex 75 10/300 GL column in a fashion almost identical to that of SOD1 under the same conditions. For both proteins, a Tris-HCl 50 mM buffer pH 8 was used. Analysis conditions: 4 °C, buffer flow rate: 0.5 mL/min.
